# Mitochondria-Mediated Apoptosis of HCC Cells Triggered by Knockdown of Glutamate Dehydrogenase 1: Perspective for Its Inhibition through Quercetin and Permethylated Anigopreissin A

**DOI:** 10.3390/biomedicines9111664

**Published:** 2021-11-11

**Authors:** Michela Marsico, Anna Santarsiero, Ilaria Pappalardo, Paolo Convertini, Lucia Chiummiento, Alessandra Sardone, Maria Antonietta Di Noia, Vittoria Infantino, Simona Todisco

**Affiliations:** 1 Department of Science, University of Basilicata, Viale dell’Ateneo lucano 10, 85100 Potenza, Italy; michelamarsico@libero.it (M.M.); anna.santarsiero@unibas.it (A.S.); ilaria.pappalardo@unibas.it (I.P.); paolo.convertini@gmail.com (P.C.); lucia.chiummiento@unibas.it (L.C.); ales.sardone95@gmail.com (A.S.); 2 Department of Biosciences, Biotechnologies and Biopharmaceutics, University of Bari, Via Orabona 4, 70125 Bari, Italy; maria.dinoia@uniba.it

**Keywords:** hGDH1, HCC, *GLUD1*, redox homeostasis, mitochondrial mass, apoptosis, inhibition, quercetin, Permethylated Anigopreissin A (PAA)

## Abstract

Metabolic reprogramming is a hallmark of cancer cells required to ensure high energy needs and the maintenance of redox balance. A relevant metabolic change of cancer cell bioenergetics is the increase in glutamine metabolism. Hepatocellular carcinoma (HCC), one of the most lethal cancer and which requires the continuous development of new therapeutic strategies, shows an up-regulation of human glutamate dehydrogenase 1 (hGDH1). GDH1 function may be relevant in cancer cells (or HCC) to drive the glutamine catabolism from L-glutamate towards the synthesis of α-ketoglutarate (α-KG), thus supplying key tricarboxylic acid cycle (TCA cycle) metabolites. Here, the effects of h*GLUD1* gene silencing (si*GLUD1*) and GDH1 inhibition were evaluated. Our results demonstrate that si*GLUD1* in HepG2 cells induces a significant reduction in cell proliferation (58.8% ± 10.63%), a decrease in BCL2 expression levels, mitochondrial mass (75% ± 5.89%), mitochondrial membrane potential (30% ± 7.06%), and a significant increase in mitochondrial superoxide anion (25% ± 6.55%) compared to control/untreated cells. The inhibition strategy leads us to identify two possible inhibitors of hGDH1: quercetin and Permethylated Anigopreissin A (PAA). These findings suggest that hGDH1 could be a potential candidate target to impair the metabolic reprogramming of HCC cells.

## 1. Introduction

Glutamate dehydrogenase (GDH) belongs to the class of oxidoreductases and catalyzes the reversible reaction of oxidative deamination of L-glutamate to α-ketoglutarate (α-KG) and ammonia, by using NAD(P)^+^ as cofactor.

Human GDH (hGDH) is a homohexamer, essentially composed of two trimers, in which each subunit is composed of about 500 aminoacidic residues and consists of three domains: the hexamer core, located near the dimer interface, the NAD binding domain, and the regulatory region of the mammalian enzyme, constituted by an antenna, a characteristic element of mammalian GDH [1] which protrudes from the NAD binding domain, and by a pivot helix [2].

Since the involvement of the hGDH is in many cellular processes, including the interconnection between the nitrogen and carbon metabolism, its activity is strictly regulated by different compounds such as ADP, ATP, GTP, L-leucine, and palmitoyl-CoA [3].

In humans, there are two isoforms: hGDH1, encoded by *GLUD1* gene located on chromosome 10, and expressed in all tissues, but at the highest levels in the liver, and hGDH2, encoded by the *GLUD2* intronless gene located on the X chromosome, and specifically expressed in neural and testicular tissues while it is almost absent in the liver [4].

Both isoforms have a high similarity in the amino acid sequence (97%) and are mainly located in the mitochondrial matrix, but have also been found in the cytoplasm and in the endoplasmic reticulus [5], the function of which is not yet known [3]. In the liver, the expression of hGDH1 is notably higher than in other tissues [3] and it works in the direction of oxidative deamination of L-glutamate to supply ammonium and L-aspartate to the urea cycle.

In tumor cells, the bioenergetic balance alteration, associated with a high demand for nutrients and oxidative stress, is the basis of the metabolic reprogramming. Beyond an increase in glycolytic levels, glutaminolysis sustains the growth and the proliferation of cancer cells. Therefore, cultured cancer cells can require up to 100-fold molar excess of L-glutamine compared to other amino acids [6], and deprivation of L-glutamine leads to growth arrest and cell death.

The upregulation of hGDH1 in different cancers such as gliomas, leukemias, liver, breast, lung, and colorectal cancers [3] could control the intracellular levels of α-KG. Through this metabolite, GDH1 provides some substrates to biosynthetic pathways, as lipid synthesis, and antioxidants, as glutathione (GSH) [7], thus supplying fumarate, which in turn activates glutathione peroxidase 1, regulating redox homeostasis and tumor growth [8]. Among the cancer types, hepatocellular carcinoma (HCC) is the most common primary liver cancers and one of the most lethal cancers. It is characterized by altered metabolic pathways and epigenetic reprogramming, in which mitochondria have an important role for the tumor cell growth and proliferation. Hallmarks are a widespread dysregulation of tricarboxylic acid (TCA) cycle and related enzymes [9] and the movement of metabolites across the inner mitochondrial membrane that is necessary to guarantee the de novo synthesis of macromolecules for cell growth [6]. Noteworthy, citrate is required for lipid biosynthesis and protein acetylation via the citrate carrier (CIC), while L-aspartate and L-glutamate, via aspartate/glutamate carrier 1 (AGC1), are needed for nucleotide biosynthesis [10,11,12]. In HCC, the role of glutamine has not yet been well established and some data indicate that L-glutamine is metabolized via glutaminase (GLS) and GDH in α-KG [13], while other data indicate a downregulation of glutamine transformation into α-KG in HCC compared to normal cells [14].

Since the inhibition of hGDH1 by shRNA or specific inhibitors impairs cell proliferation and tumor growth [15,16], to investigate how this metabolic pathway contributes to HCC progression may represent a new therapeutic strategy minding hGDH as a pharmacological target.

Here, we evaluated the effect of *GLUD1* gene silencing on HCC cell proliferation, cell growth, and apoptosis activation as well as the effect of inhibition of GDH1 activity by quercetin and Permethylated Anigopreissin A (PAA).

It was reported that phenol derivatives, as Epigallocatechin gallate (EGCG) [17] and chlorogenic acid [18] or synthetic organoselenium compounds, as Ebselen and Propylselen [19] inhibited hGDH.

Quercetin is a flavonoid abundantly present in several food as onions, apples, red grapes, broccoli, as well as tea and red wine, with anti-inflammatory, antitumor, antidiabetic activities [20,21], as well as with antioxidant properties related to its ability to regulate cellular glutathione levels, to inhibit the activity of enzymes with oxidative properties, and to remove reactive oxygen species (ROS) [22].

PAA is a synthetic derivative of Anigopreissin A [23], a dimer of resveratrol isolated from the root of *Anigozanthos preissii, Musa Cavendish* rhizomes, and *Macropidia fuliginosa bulbs*, which has shown a selective cytotoxicity for HCC cells [24]. Both compounds are able to selectively act on tumor cells by activating an apoptotic mechanism and showing mainly effects at mitochondrial levels [24,25]. Starting from these considerations, we evaluated if GDH1 could be a target of quercetin and PAA and if its activity could be modulated from these two compounds as a future strategy for HCC treatment.

## 2. Materials and Methods

In Supplementary materials, the Research Design to define the research plan of the described experimental procedures is provided.

### 2.1. Cell Culture

HepG2 hepatic cancer cell line was purchased from the European Type Culture Collection. HepG2 cells were cultured in Dulbecco’s modified Eagle’s medium (DMEM) supplemented with 10% (*v*/*v*) fetal bovine serum, 2 mM L-glutamine and 1% penicillin/streptomycin (Sigma-Aldrich, St Louis, MO, USA). Human hepatocytes (HH, Lonza, Walkersville, MD, USA) were grown in hepatocyte culture medium (Lonza) as per manufacturer’s instructions. All cells were maintained at 37 °C in 5% CO_2_ in a water-saturated atmosphere.

### 2.2. GLUD1 Expression Analysis

Gene-expression analyses of *GLUD1* (UniProtKB-P00367, DHE3_HUMAN, https://www.uniprot.org/uniprot/P00367#expression, assessed on 18 September 2021) were carried out by Human Protein Atlas (website https://www.proteinatlas.org, assessed on 18 September 2021) and Genevisible (https://genevisible.com/search, assessed on 18 September 2021).

Human Protein Atlas is a database that collects all the available information on human proteins using various techniques that include imaging, proteomic, and transcriptomic analysis obtained by microarray with antibodies and massive RNA sequencing. The analysis was inferred in the different tissues and cell subtypes by using the keyword “*GLUD1*” https://www.proteinatlas.org/ENSG00000148672-GLUD1 (assessed on 18 September 2021). The data relating to the expression in the various tissues were acquired at this link https://www.proteinatlas.org/ENSG00000148672-GLUD1/celltype (assessed on 18 September 2021), while those relating to expression in cell lines, were extrapolated from this link https://www.proteinatlas.org/ENSG00000148672-GLUD1/cell (website: https://www.proteinatlas.org/, assessed on 18 September 2021).

The average expression level is reported as NX normalized value, where a NX value of 1,0 is the threshold value for the expression of the corresponding protein.

Genevisible (https://genevisible.com/search, assessed on 18 September 2021) is a search portal based on normalized and curated expression data from the Genevestigator software. Data collected in the database comes from the Affymetrix Human Genome U133 Plus 2.0 Array platform. The search was performed by searching for “*GLUD1*”, “LIVER” and “*Homo sapiens*” as keywords. The data relating to “Liver” are present at the link https://genevisible.com/tissues/HS/Gene%20Symbol/GLUD1 (assessed on 18 September 2021), those relating to “Cancer liver” at the link https://genevisible.com/cancers/HS/Gene%20Symbol/GLUD1 (assessed on 18 September 2021), and those relating to “Hepatocyte (ESC)” and “Hepatocyte-immature (WA09)” at the link https://genevisible.com/cell-lines/HS/Gene%20Symbol/GLUD1 (assessed on 18 September 2021). The average expression level of *GLUD1* gene is reported on a logarithmic scale (log2 scale) and the number of samples under examination is indicated.

### 2.3. Transfection of GLUD1 siRNA

The *GLUD1* human gene was transiently silenced by RNA interference experiments. To this end, HepG2 or HH cells were transfected for two consecutive days with a specific small interfering RNA (siRNA) targeting *GLUD1* (s13, Silencer^®^ Select Validated, Life Technologies, Paisley, UK), or control scramble siRNA (4390843, Thermo Fisher Scientific, Waltham, MA, USA) using Lipofectamine RNAiMax Reagent (13778030, Thermo Fisher Scientific).

### 2.4. Cell Proliferation Assay

The effect of *GLUD1* gene silencing on cell proliferation was evaluated by CellTiter-Glo^®^ 2.0 Cell Viability Assay (G9242 Promega, Madison, WI, USA) according to the manufacture’s protocol. Briefly, HepG2 cells were plated at 4 × 10^3^ cells/well and HH at 2 × 10^3^ cells/well in a 96-well plate and incubated overnight. The day after HepG2 and HH cells were transfected with siRNA targeting human *GLUD1* (si*GLUD1*) or control scramble siRNA (C). Seventy-two hours later, CellTiter-Glo Luminescence stain was added and the luminescence signal was read by the plate reader (GloMax, Promega) [26].

### 2.5. Caspase Assay

HepG2 cells were seeded in a 96-well plate at a density of 2 × 10^4^ cells per well, and after 24 h, were transfected with siRNA targeting human *GLUD1* or control scramble siRNA. Twenty-four hours later, the medium was removed and the caspase 3/7 or 9 activity was determined using a luminescent Caspase-Glo^®^ 3/7 or 9 assay kit, as described previously [24]. The luminescence was measured using GloMax plate reader.

### 2.6. DNA Fragmentation Assay

On *GLUD1* silencing-HepG2 cells, nuclear morphology was assessed using 4′,6-diamidino-2-phenylin-dole (DAPI) staining, a DNA-specific probe which forms a fluorescent complex by attaching in the minor grove of A-T rich sequences of DNA as described in [24]. The appropriate excitation and emission wavelengths were shown to be 360 nm and 450 nm, respectively. Cells were washed with PBS and treated with 300 nM DAPI for 10 min. Nuclear fragmentation was examined with Evos Floid Cell Imaging Station (magnification 20×). Images are representative of three independent experiments.

### 2.7. Real-Time PCR

Total RNA was extracted from HepG2 cells by RNeasy Mini Kit (Qiagen, Venlo, The Netherlands). cDNA was obtained by using GeneAmp RNA PCR Core kit (Life technologies) according to the manufactures’ instruction. Real-time PCR was performed by using specific TaqMan Gene Expression Assays for human *GLUD1* (Hs03989560_s1), BCL2 (Hs00608023_m1), BAX (Hs00180269_m1) and β-actin (4326315E) were purchased from Life technologies. BCL2 and BAX transcript levels were normalized against β-actin expression levels. Data were analyzed according to the ΔΔct method [27].

### 2.8. Mitochondrial Mass Analysis

MitoTracker^®^ Green FM (MTG, Thermo Fisher Scientific), a green-fluorescent mitochondrial stain which appears to localize to mitochondria regardless of mitochondrial membrane potential, was used to examine changes in mitochondrial mass following the manufacturer’s instructions. Briefly, HepG2 cells were seeded in a 24-well plate at a density of 2 × 10^5^ cells per well, and after 24 h were transfected with si*GLUD1* or control scramble siRNA (C). The day after, the medium was removed and added prewarmed (37 °C) staining solution containing 25 nM MitoTracker^®^ probe. After 30 min of incubation at 37 °C in the dark, cells were washed with PBS and analyzed by Evos Floid Cell Imaging Station (magnification 20×). MTG has excitation and emission peaks at 490 and 516 nm, respectively. Images are representative of three independent experiments. For a quantitative analysis, the fluorescence was also measured (Ex/Em 490/516 nm) by using a GloMax plate reader (Promega).

### 2.9. Mitochondrial Membrane Potential Analysis

MitoTracker™ Red CMXRos (MTR, Thermo Fisher Scientific), a red-fluorescent dye that stains mitochondria in live cells and its accumulation is dependent upon membrane potential, was used to examine changes in mitochondrial membrane potential as described in [24]. In brief, HepG2 cells were seeded and transfected as previously described. After 24 h of gene silencing, the medium was removed and cells were staining with a prewarmed (37 °C) 25 nM MitoTracker™ probe for 30 min at 37 °C in the dark. Then, cells were washed with PBS and analyzed by Evos Floid Cell Imaging Station (magnification 20×). MTR has excitation and emission peaks at 579 and 599 nm, respectively. Image analysis was performed with ImageJ to quantify the intensity of mitochondrial red within the silenced and control cells. Images are representative of three independent experiments. For a quantitative analysis of the mitochondrial membrane potential, the fluorescence was also measured (Ex/Em 579/599 nm) by using a GloMax plate reader (Promega).

### 2.10. Mitochondrial Superoxide Analysis

MitoSOX™ Red mitochondrial superoxide indicator (MS, Thermo Fisher Scientific), a novel fluorogenic dye specifically targeted to mitochondria in live cells, was used to detect mitochondrial ROS, especially superoxide, following the manufacturer’s instructions. In detail, HepG2 cells were seeded and transfected as previously described. Cells were staining with 5 μM MitoSOX™ reagent solution for 45 min at 37 °C in the dark. After incubation, HepG2 cells were washed with PBS and analyzed by Evos Floid Cell Imaging Station (magnification 20×). MS has excitation and emission peaks at 510 and 580 nm, respectively [28]. Image analysis was performed with ImageJ to quantify the intensity of mitochondrial red within the silenced and control cells. A quantitative analysis of the mitochondrial superoxide levels was obtained by using a GloMax plate reader (Promega) at 510/580 nm. Images are representative of three independent experiments.

### 2.11. Purified Bovine Liver GDH Activity

GDH activity was determined spectrophotometrically in the direction of reductive amination using ammonia and α-KG as substrates [29]. Purified GDH1 from bovine liver (Sigma Aldrich, Type II from bovine liver with a specific activity of 40 units/mg of protein) was used in a mixture of reaction containing Tris HCl pH 7.4, 100 mM NH_4_Cl, 0.4 mM NADH, α-KG in Tris HCl in a wide range of concentrations (0.6, 1.5, 2.5, 4, and 8 mM) until a final volume of 250 μL. The reaction was evaluated on a 96-well plate using a Thermo Scientific Multiskan spectrophotometer by monitoring the decrease of NADH absorption at 340 nm for 5 min. For the inhibition, two sets of experiments were performed in the absence or presence of quercetin and PAA (2, 4, 8, 12, 16 µM). Purified bovine liver GDH was used at 0.3 U per 250 μL reaction and GDH activity was calculated using an extinction coefficient of 6.22 mM^−1^ cm^−1^.

### 2.12. hGDH Activity in Cellular Extracts

The activity of hGDH, in the presence or absence of quercetin and PAA (2, 4, 8 or 12 µM), was evaluated in cellular extracts of HepG2 and HH, as described in [21].

Briefly, 1 × 10^7^ cells were washed twice in ice-cold PBS. Cell pellets were collected and then lysed with freeze and thaw cycles. After centrifugation, the supernatant was collected and the protein concentration was determined by Bradford assay. 150 μg of cellular extract was added to a mixture of reaction containing Tris HCl pH 7.4, 100 mM NH_4_Cl, 0.4 mM NADH, 2.5 mM α-KG until a final volume of 250 μL. The reaction was evaluated on a 96-well plate using a Thermo Scientific Multiskan spectrophotometer by monitoring the decrease of NADH absorption at 340 nm for 10 min. For the inhibition, two sets of experiments were performed in the absence or presence of quercetin and PAA (4, 8 µM).

### 2.13. Statistical Analysis

Data are presented as mean values ± standard deviation (SD) of, at least, three independent experiments run in triplicate. Statistical analysis was performed using Student’s t-test or one-way ANOVA [30,31]. According to the *p*-value, differences were considered as significant (*p* < 0.05) or highly significant (*p* < 0.001).

## 3. Results

### 3.1. GLUD1 Gene Expression

**Figure 1:**
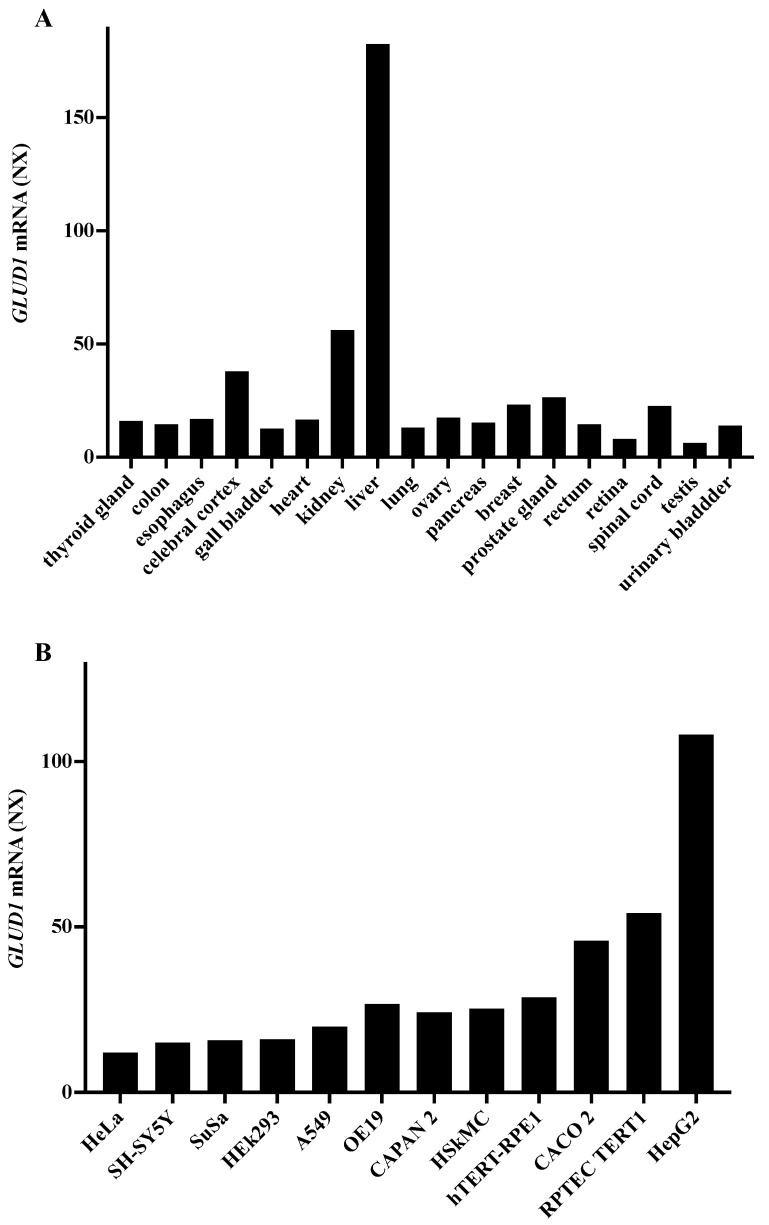
*GLUD1* mRNA expression: The analysis of mRNA expression profile of *GLUD1* gene in different tissues (**A**) and cell lines (**B**) was obtained by consulting the database Human Protein Atlas. The keyword used to start the search was “*GLUD1*” https://www.proteinatlas.org/ENSG00000148672-GLUD1 (assessed on 18 September 2021). The data relating to the expression in the various tissues shown in A were acquired at this link https://www.proteinatlas.org/ENSG00000148672-GLUD1/celltype (assessed on 18 September 2021), while the data relating to expression in cell lines, (**B**), were extrapolated from this link https://www.proteinatlas.org/ENSG00000148672-GLUD1/cell (website: https://www.proteinatlas.org/, assessed on 18 September 2021). The data are reported as NX normalized values, where a NX value of 1.0 is the threshold value for the expression of the corresponding protein.

By analyzing the database Expression Atlas, *GLUD1* can be considered a housekeeping gene because the mRNA is found expressed in all human tissues. Interestingly, the highest expression levels are displayed in the liver (Figure 1A) suggesting a high hepatic activity of hGDH1 which represents an important step to eliminate the excess of nitrogen and ammonia derived from protein metabolism via the urea cycle in the human body. The analysis of *GLUD1* mRNA expression levels in cell lines highlights the significantly higher expression of this gene in HepG2 cells than in the other tumor cell lines (Figure 1B). Moreover, by using Genevisible database, it has been possible to perform a comparison between *GLUD1* gene expression in normal and cancer livers. These data show a double amount of *GLUD1* mRNA in cancer liver compared to both normal liver and normal hepatocytes (Table 1).

### 3.2. Cytotoxic Effect of GLUD1 Gene Silencing on HCC and HH Cells

**Figure 2:**
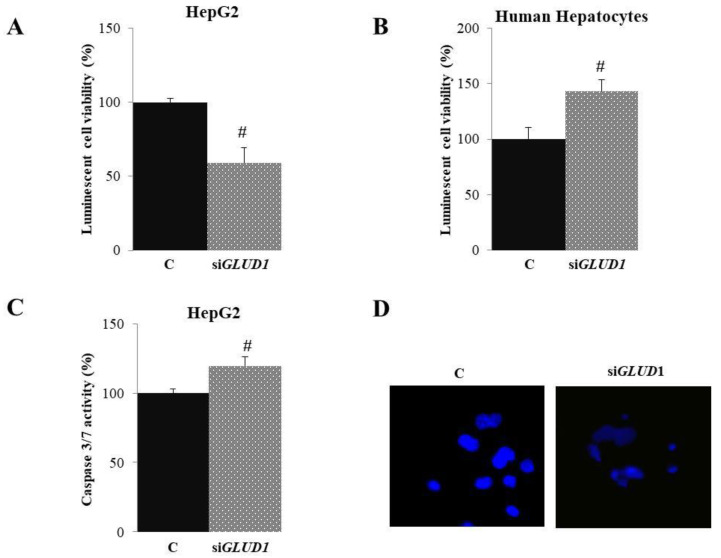
Apoptotic effect of *GLUD1* siRNA. HepG2 and Human Hepatocytes were transfected with siRNA targeting human *GLUD1* (si*GLUD1*) or control scramble siRNA (**C**). (**A**,**B**) Seventy-two hours later, cell viability was assessed by using Promega CellTiter-Glo^®^ 2.0 Assay. (**C**) Twenty-four hours after *GLUD1* gene silencing, HepG2 cells were assayed for caspase 3/7 and (**D**) stained with DAPI followed by visualization under fluorescence microscope (magnification 20×). Images in (**D**) are representative of three independent experiments with similar results. In (**A**–**C**), means ± S.D. of four replicate independent experiments are shown; differences between samples and relative controls are significant (# *p* < 0.05, Student’s *t*-test).

First, we investigated the cytotoxic activity of *GLUD1* gene silencing on HepG2 and HH cells by using Promega CellTiter-Glo^®^ 2.0 Assay after ascertaining the good efficiency of *GLUD1* gene silencing (Figure S1). To this end, HepG2 and HH cells were transfected with siRNA targeting the human *GLUD1* gene (si*GLUD1*) or control scramble siRNA (C). After 72 h, CellTiter-Glo Luminescence stain was added and the luminescence signal was read by GloMax plate reader. As shown in Figure 2A, there was a significant reduction of HepG2 cell proliferation of about 50% when *GLUD1* gene was silenced compared to control cells. Interestingly, *GLUD1* gene silencing did not lead to a cytotoxic effect in human hepatocytes; indeed, a significant increase in cell proliferation was observed (Figure 2B).

### 3.3. Apoptotic Activity in GLUD1-Silenced HCC Cells

Since we observed a reduction in proliferation in HepG2 following the silencing of the *GLUD1* gene, we decided to analyze the proteins linked to cell death by apoptosis. Caspase family members are at the nexus of critical regulatory networks controlling cell death and inflammation. Although caspases are most often associated with apoptosis, there has been persistent evidence that some of these enzymes can also affect proliferation [32]. Caspases 3/7 in apoptotic cells are activated extrinsically (ligand of death, usually on the surface of the cytoplasmic membrane) and intrinsically (mitochondrial, by the apoptosome complex) [33] Then, we tested HepG2 cells for caspase 3/7 activity. To this end, we silenced *GLUD1* gene in HepG2 cells and we measured effect on caspase 3/7 activity by Caspase-Glo^®^ 3/7 luminescence assay. Figure 2C shows that the activation of caspases by 19.8 ± 6.15 occurred in HepG2 cells after *GLUD1* gene silencing. To evaluate changes in nuclear morphology after *GLUD1* gene silencing, we stained cells with DNA-binding DAPI and visualized them under a fluorescence microscope. Interestingly, in agreement with cell viability data, gene silencing induced significant nuclear morphological changes with less intense DAPI staining and less defined nuclear structures than unsilenced control cells (Figure 2D). Changes in nuclear morphology following *GLUD1* silencing suggest that cells are undergoing apoptosis. Taken together, these results indicate that the cytotoxic effect of *GLUD1* gene silencing in HepG2 cells is mediated by the induction of apoptosis.

### 3.4. GLUD1 Gene Silencing Induces Apoptosis via the Intrinsic Pathway in HCC Cells

**Figure 3:**
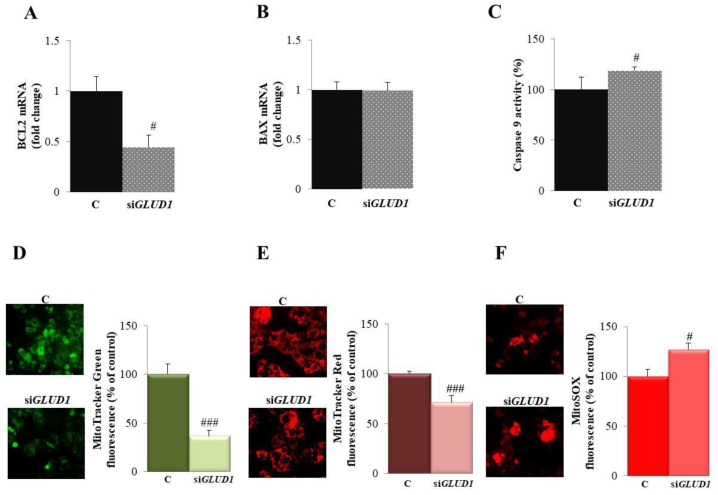
*GLUD1* gene silencing induces apoptosis via the mitochondrial pathway. Total RNA extracted from HepG2 cells transfected with si*GLUD1* (si*GLUD1*) or control scramble siRNA (**C**) was used to quantify BCL2 (**A**) and BAX (**B**) mRNA levels. (**C**) HepG2 cells transfected as in (**A**,**B**) were assayed for caspase 9 activity. (**D**–**F**) HepG2 cells transfected as in (**A**,**B**) were stained with MitoTracker Green FM (**D**), MitoTracker™ Red CMXRos (**E**), and MitoSOX Red Mitochondrial Superoxide Indicator (**F**) and visualized under fluorescence microscope (magnification 20×). Images and relative bar graphs in (**D**–**F**) are representative of three independent experiments with similar results. In (**A**–**C**), mean values ± SD of three independent experiments performed in triplicate are shown; where indicated differences between samples and relative controls are significant (# *p* < 0.05, ### *p* < 0.001, Student’s *t*-test).

Taking into consideration that *GLUD1* gene silencing leads to the apoptosis of HepG2 cells, we wondered if the activation mechanism was the intrinsic pathway since it is the most frequently deregulated type of cell death in cancers [34]. Therefore, we evaluated the mRNA expression of two members of the BCL2 family: pro-apoptotic BAX and anti-apoptotic BCL2, whose balance can dictate the cellular fate since they act as critical life-death decision points in the apoptotic process [35]. When the specific siRNA targeting human *GLUD1* was employed, BCL2 mRNA levels were reduced by more than half in HepG2 cells (Figure 3A), while the expression of BAX gene was not affected (Figure 3B). BCL2 downregulation is responsible for mitochondrial changes closely related to the intrinsic pathway of apoptosis [36]. As a consequence of BCL2 downregulation, we observed an increased activation of caspase 9: when *GLUD1* was silenced, caspase 9 activity raised of about 20% (Figure 3C). To prove more strongly the involvement of the mitochondrial pathway in *GLUD1* gene silencing-induced apoptosis, we performed staining with different fluorogenic dyes assessing the mitochondrial mass, the membrane potential and the oxidative stress. When HepG2 cells were stained with MitoTracker Green, we observed a 75% ± 5.89% decrease in its intensity when *GLUD1* was silenced (Figure 3D) that may indicate the degradation of mitochondria since this probe is used to quantify the mitochondrial mass regardless of mitochondrial membrane potential [37]. Similar results were obtained when the membrane potential was analyzed by using MitoTracker Red CMXRos, even if the decrease was weaker (Figure 3E). On the other hand, when we assessed the effect of *GLUD1* gene silencing on mitochondrial oxidative stress and production of superoxide radicals, we observed a significant increase in mitochondrial superoxide anion production, detected as an increase in the fluorescence of the mitochondria-targeted fluorochrome MitoSOX (Figure 3F). Overall findings suggest that *GLUD1* gene silencing activates the intrinsic pathway of apoptosis.

### 3.5. Quercetin and PAA Inhibit GDH Activity

The above results demonstrated the involvement of hGDH1 in cell proliferation and growth of HepG2 cells by suggesting that glutamate dehydrogenase may be a potential candidate target for HCC. We therefore analyzed the effect of two possible inhibitors on the activity of GDH: quercetin and PAA.

Firstly, we evaluated the activity of purified GDH1 from bovine liver. The reaction of reductive amination of α-KG was estimated by measuring the absorbance variation of NADH in the presence and absence of different concentrations of quercetin and PAA. GDH1 activity in the presence of EGCG was also measured.

**Figure 4:**
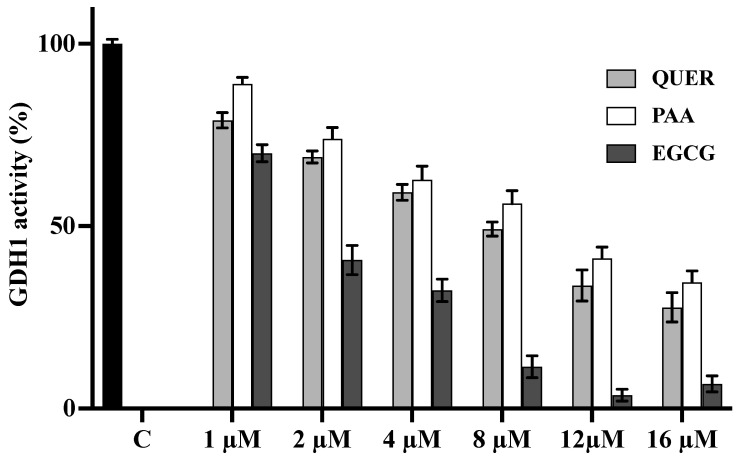
Effect of Quercetin, PAA, and EGCG on the activity of purified bovine liver GDH1. The activity of purified bovine liver GDH1 was initiated by adding NH_4_Cl in the absence (black column) or in the presence of increasing external concentrations of Quercetin (light grey columns), PAA (white columns) or EGCG (grey columns). The residual activities (%) in presence of quercetin, PAA or EGCG are the means ± SD of at least three independent experiments. Differences between samples are significant (*p* < 0.05, one-way ANOVA).

All tested compounds inhibited GDH1 activity in a concentration-dependent manner in a range of 1–16 μM (Figure 4). A residual activity of about 50% was observed in the presence of 8 μM of quercetin and 12 μM of PAA, respectively, whereas 2 μM of EGCG have shown an inhibition of about 50%. In the presence of 1 μM of quercetin and PAA, the inhibition shown by each compound was very low (residual activity of about 80–90% for quercetin and PAA, respectively).

**Figure 5:**
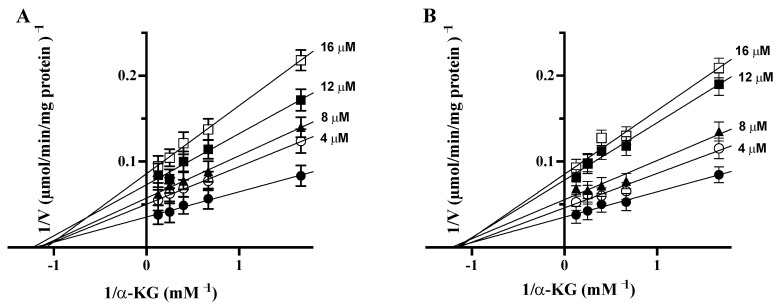
Lineweaver–Burk plot reporting the purified bovine liver GDH activity at the indicated concentrations of α-KG in the absence (●) or in the presence of 4(○), 8 (▲), 12 (■), and 16 (□) μM of PAA (**A**) and quercetin (**B**). The values shown are the means ± SD for three independent measurements.

To elucidate the inhibition mechanism, double-reciprocal plots were constructed from the reciprocal of initial rates versus the reciprocal of α-KG concentrations with or without different concentrations of PAA or quercetin (Figure 5A,B). The Michaelis–Menten (half-saturation) constant (Km) of the purified bovine liver GDH1 was determined by measuring the initial rate by varying the α-KG concentrations in the presence of a fixed saturating concentration of ammonia and NADH. The Km value was 0.84 ± 0.02 mM and the Vmax was 28.27 ± 1.03 μmol/min/mg protein for α-KG, in agreement with reported data [38].

The results of double-reciprocal plots revealed that both quercetin and PAA are no competitive inhibitors for α-KG, because we observed a decrease of Vmax and no change in Km in the presence of quercetin and PAA at different tested concentrations (4, 8, 12, and 16 μM).

Secondary plots of the slopes and intercepts against the reciprocals of the concentrations of each inhibitor were used to determine the inhibitor constants, Ki and Kii, and for no competitive inhibitors Ki = Kii. For quercetin and PAA, secondary plots were built by using four concentrations (4; 8; 12 and 16 μM) of quercetin and PAA (data not shown). The Ki and Kii values for quercetin were 9.2 and 9.6 μM, respectively, and for PAA are 10 and 10.5 μM, respectively, confirming that both molecules are no competitive inhibitors of purified liver bovine GDH1.

**Figure 6:**
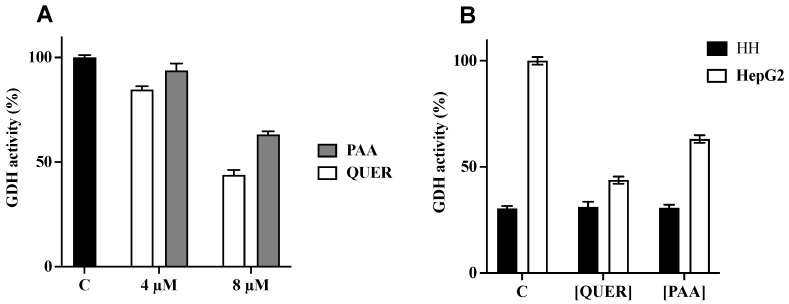
Inhibition of hGDH activity: (**A**) The activity of GDH was evaluated on cellular extract of HepG2 cells. The reaction was started by adding NH_4_Cl without (black column) or with 4 and 8 μM of Quercetin (QUER, white columns) or PAA (grey columns). The residual activities (%) in the presence of quercetin or PAA are the means ± SD of at least three independent experiments. (**B**) Effect of Quercetin and PAA on activity of GDH was evaluated on cellular extract of HepG2 (white columns) or Human Hepatocyte (HH) (black columns). The reaction was started by adding NH_4_Cl without or with 8 μM of Quercetin (QUER) or PAA. The residual activities (%) in the presence of quercetin or PAA are the means ± SD of four independent experiments. Differences between samples are significant (*p* < 0.05, one-way ANOVA). Abbreviations: PAA: Permethylated anigopreissin A, QUER: Quercetin, HH: Human Hepatocytes.

Finally, we evaluated the inhibition of human GDH on cellular extracts of HepG2 and HH. The GDH activity of HepG2 cellular extract was determined in the presence of 4 and 8 μM quercetin or PAA (Figure 6A). The inhibition was about 56% and 37% in the presence of 8 μM quercetin or PAA, respectively, compared to control in the absence of inhibitors. In the same conditions, no inhibition was detected for both inhibitors (8 μM) on cellular extract of HH since GDH activity was lower in the HH cell extract than in the HepG2 cell extract (Figure 6B). Altogether, our data point out PAA and quercetin as inhibitors of GDH1 activity in HCC.

## 4. Discussion

Cancer cells are characterized by high proliferation sustained by metabolic reprogramming ensuring increased energy requires and maintenance of redox balance [6].

Glutaminolysis is a main metabolic pathway for this adaptation process, because L-glutamine is a good substrate for the mitochondrial oxidation of cancer cells [39] by suggesting that alterations in the glutamine pathway might control tumor growth. HCC pathogenesis is a multistep process and altered glutaminolysis pathway is highlighted as a hallmark of cancer. Glutamine catabolism includes GLS and GDH or amino acid transaminases [40].

hGDH1 is found up-regulated in many cancer cells producing α-KG, where it replenishes intermediates of the TCA cycle as biosynthetic precursors, particularly in proliferating cells [40].

In this study, we demonstrate that human *GLUD1* gene silencing in HCC cells reduces HepG2 cell proliferation, while it has an opposite effect in normal hepatocytes as well as previously demonstrated in other cancer cells [8]. Moreover, *GLUD1*-silenced HepG2 cells show an activation of caspases 3/7 and significant nuclear morphological changes. Apoptosis activation takes place through the intrinsic pathway, with a decrease in BCL2 mRNA levels and a significant reduction in mitochondrial membrane potential.

Interestingly, *GLUD1* knockdown also generates a boost in mitochondrial superoxide anion production. These outcomes provide evidence for the involvement of hGDH1 in promoting cell proliferation and in ensuring mitochondrial functionality of HCC cells.

Therefore, GDH1 activity seems relevant in maintaining redox homeostasis, in agreement with previous investigations reporting that *GLUD1* gene silencing resulted in a decrease of cell proliferation and an increase of mitochondrial ROS levels in breast, lung, and glia cancer cells [8,16].

In fact, a moderate increase in ROS levels in many types of cancer cells when compared to normal cells is functional to promote cell growth and proliferation, and the maintenance of ROS homeostasis is ensured by various systems as glutathione peroxidase (GPx), gluthathione reductase (GR), thioredoxin (Trx), superoxide dismutases (SOD), catalase (CAT), and peroxiredoxin (PRX) [8].

Furthermore, we provide evidence for a modulation of mitochondrial biogenesis-increased in HCC [41] through GDH1 function, since a decrease in mitochondrial mass has been observed in *GLUD1*-silenced HCC cells.

These findings point out the crucial role of hGDH1 working as an important crossroad in HCC cells. Indeed, by providing α-KG, hGDH1 may sustain the TCA cycle in direct and reverse mode-depending on cellular conditions, thus supporting mitochondrial energetics and mitochondrial function.

In fact, glutamate-deriving α-KG is channeled, via α-KG dehydrogenase complex (KGDHC), in the TCA cycle providing intermediates for biosynthetic and energetic needs as well as the proto-oncometabolite fumarate which leads to activation of GPx1, involved in redox homeostasis [8]. Moreover, in fumarate hydratase (FH)-deficient cancer cells, accumulated fumarate binds to the glutathione to produce succinated glutathione (GSF). GSF enhances mitochondrial ROS and HIF-1 activation. In addition, increased ROS levels also correlate with epigenetic dysregulations of these cells [42].

Alternatively, in hypoxic conditions, in which cancer cells rewire many mitochondrial processes [43], α-KG, produced by hGDH, [44] might be reductively carboxylated to isocitrate by mitochondrial isocitrate dehydrogenase (IDH2) in a reverse mode of TCA cycle function, fostering citrate synthesis for fatty acid biosynthesis This citrate synthesis requires a high mitochondrial NADPH/NADP^+^ ratio ensured by GDH activity and mitochondrial nicotinamide nucleotide transhydrogenase that synthesizes NADPH from NADH. In addition, in cancer cells with defective FH, α-KG is concomitantly metabolized in both pathways: oxidative decarboxylation via the TCA cycle and reductive carboxylation via IDH2 [45]. It is possible to speculate that in these conditions, GDH activity functions to produce an overflow of α-KG to ensure high levels of reducing equivalents used to synthesize isocitrate to sustain the synthesis of citrate.

In this context, the identification of possible inhibitors of hGDH1 could be a strategy to modulate cancer cell growth and proliferation. Few compounds have been reported as inhibitors of hGDH among which phenol derivatives, as Epigallocatechin gallate (EGCG) [17] and chlorogenic acid [18], or Ebselen and Propylselen [16,19].

Here, the effects of quercetin and PAA on the activity of purified bovine liver GDH1 and HepG2 extracts were tested. Both compounds have previously shown cytotoxic and antiproliferative effects on cancer cells. Furthermore, quercetin as well as PAA work mainly by mitochondrial targeting through the activation of the intrinsic apoptotic pathway [24,25,46].

Our results described here demonstrate that quercetin and PAA are no competitive inhibitors of purified bovine liver GDH1 and are able to inhibit hGDH on HCC cellular extracts. For this reason, they can be considered as good candidates for hGDH1 inhibition. Our findings shed more light on molecular mechanisms underlying the anti-cancer activity of quercetin in HCC. Indeed, recently, antineoplastic properties of quercetin have been explored beyond its antioxidant effects in HCC. These investigations unveiled ameliorated HCC and hepatic function after quercetin treatment. However, only specific pathways have been related to quercetin function in HCC [47].

About PAA, although it has been well demonstrated its antiproliferative effect specific for cancer cells and stronger for HCC cells, no molecular target has been identified so far. Therefore, this study suggests that PAA induces its effect in HCC cells via GDH1 inhibition, although we cannot exclude other molecular mechanisms that may be investigated in the future. Further studies are warranted to determine the effect of these two promising inhibitors in vivo.

In conclusion, the gene silencing of human *GLUD1* gene, upregulated in HCC cells, reduces HepG2 cell proliferation without any decrease in normal hepatocyte cell growth. *GLUD1* gene silencing affects redox homeostasis and leads to mitochondrial apoptosis of HepG2, pointing out the inhibition of GDH1 activity as an interesting strategy to impair the metabolic reprogramming of HCC cells.

## Figures and Tables

**Table 1:** Expression analyses performed by Genevisible (http://genevisible.com, assessed on 18 September 2021) showing the comparison between *GLUD1* gene expression in normal and cancer liver. Hepatocyte (ESC): Hepatocytes derived from embryonic stem cells. Hepatocyte-immature (WA09): generation of hepatocyte-like cells from human embryonic stem cell line WA09.

Tissues/Cell Lines	Number of Samples	Expression Level (Log2 Scale)-Average
Liver	878	1633
Cancer liver	48	1785
Hepatocyte (ESC)	53	1618
Hepatocyte-immature (WA09)	3	16

## Data Availability

The data used to support the findings of this study are available from the corresponding authors upon request.

## Supplementary Materials

The following are available online at https://www.mdpi.com/article/10.3390/biomedicines9111664/s1. Research Design, Figure S1: *GLUD1* gene silencing in HepG2 cells.

## References

[1] Hoffpauir Z.A., Sherman E., Smith T.J. (2019). Dissecting the Antenna in Human Glutamate Dehydrogenase: Understanding Its Role in Subunit Communication and Allosteric Regulation. Biochemistry.

[2] Li M., Li C., Allen A., Stanley C.A., Smith T.J. (2011). The structure and allosteric regulation of glutamate dehydrogenase. Neurochem. Int..

[3] Plaitakis A., Kalef-Ezra E., Kotzamani D., Zaganas I., Spanaki C. (2017). The Glutamate Dehydrogenase Pathway and Its Roles in Cell and Tissue Biology in Health and Disease. Biology.

[4] Shashidharan P., Clarke D.D., Ahmed N., Moschonas N., Plaitakis A. (1997). Nerve tissue-specific human glutamate dehydrogenase that is thermolabile and highly regulated by ADP. J. Neurochem..

[5] Mathioudakis L., Bourbouli M., Daklada E., Kargatzi S., Michaelidou K., Zaganas I. (2019). Localization of Human Glutamate Dehydrogenases Provides Insights into Their Metabolic Role and Their Involvement in Disease Processes. Neurochem. Res..

[6] Todisco S., Convertini P., Iacobazzi V., Infantino V. (2019). TCA Cycle Rewiring as Emerging Metabolic Signature of Hepatocellular Carcinoma. Cancers.

[7] Chen J.-Q., Russo J. (2012). Dysregulation of glucose transport, glycolysis, TCA cycle and glutaminolysis by oncogenes and tumor suppressors in cancer cells. Biochim. Biophys. Acta.

[8] Jin L., Li D., Alesi G.N., Fan J., Kang H.-B., Lu Z., Boggon T.J., Jin P., Yi H., Wright E.R. (2015). Glutamate Dehydrogenase 1 Signals through Antioxidant Glutathione Peroxidase 1 to Regulate Redox Homeostasis and Tumor Growth. Cancer Cell.

[9] Wallace D.C. (2012). Mitochondria and cancer. Nat. Rev. Cancer.

[10] Infantino V., Dituri F., Convertini P., Santarsiero A., Palmieri F., Todisco S., Mancarella S., Giannelli G., Iacobazzi V. (2019). Epigenetic upregulation and functional role of the mitochondrial aspartate/glutamate carrier isoform 1 in hepatocellular carcinoma. Biochim. Biophys. Acta Mol. Basis Dis..

[11] Convertini P., Todisco S., De Santis F., Pappalardo I., Iacobazzi D., Castiglione Morelli M.A., Fondufe-Mittendorf Y.N., Martelli G., Palmieri F., Infantino V. (2019). Transcriptional Regulation Factors of the Human Mitochondrial Aspartate/Glutamate Carrier Gene, Isoform 2 (SLC25A13): USF1 as Basal Factor and FOXA2 as Activator in Liver Cells. Int. J. Mol. Sci..

[12] Yoo H.C., Park S.J., Nam M., Kang J., Kim K., Yeo J.H., Kim J.-K., Heo Y., Lee H.S., Lee M.Y. (2020). A Variant of SLC1A5 Is a Mitochondrial Glutamine Transporter for Metabolic Reprogramming in Cancer Cells. Cell Metab..

[13] DeWaal D., Nogueira V., Terry A.R., Patra K.C., Jeon S.-M., Guzman G., Au J., Long C.P., Antoniewicz M.R., Hay N. (2018). Hexokinase-2 depletion inhibits glycolysis and induces oxidative phosphorylation in hepatocellular carcinoma and sensitizes to metformin. Nat. Commun..

[14] Björnson E., Mukhopadhyay B., Asplund A., Pristovsek N., Cinar R., Romeo S., Uhlen M., Kunos G., Nielsen J., Mardinoglu A. (2015). Stratification of Hepatocellular Carcinoma Patients Based on Acetate Utilization. Cell Rep..

[15] Liu G., Zhu J., Yu M., Cai C., Zhou Y., Yu M., Fu Z., Gong Y., Yang B., Li Y. (2015). Glutamate dehydrogenase is a novel prognostic marker and predicts metastases in colorectal cancer patients. J. Transl. Med..

[16] Zhang J., Wang G., Mao Q., Li S., Xiong W., Lin Y., Ge J. (2016). Glutamate dehydrogenase (GDH) regulates bioenergetics and redox homeostasis in human glioma. Oncotarget.

[17] Li C., Li M., Chen P., Narayan S., Matschinsky F.M., Bennett M.J., Stanley C.A., Smith T.J. (2011). Green tea polyphenols control dysregulated glutamate dehydrogenase in transgenic mice by hijacking the ADP activation site. J. Biol. Chem..

[18] Domith I., Duarte-Silva A.T., Garcia C.G., Calaza K.d.C., Paes-de-Carvalho R., Cossenza M. (2018). Chlorogenic acids inhibit glutamate dehydrogenase and decrease intracellular ATP levels in cultures of chick embryo retina cells. Biochem. Pharmacol..

[19] Hou W., Lu S., Zhao H., Yu Y., Xu H., Yu B., Su L., Lin C., Ruan B.H. (2019). Propylselen inhibits cancer cell growth by targeting glutamate dehydrogenase at the NADP+ binding site. Biochem. Biophys. Res. Commun..

[20] Ezzati M., Yousefi B., Velaei K., Safa A. (2020). A review on anti-cancer properties of Quercetin in breast cancer. Life Sci..

[21] Santarsiero A., Convertini P., Vassallo A., Santoro V., Todisco S., Iacobazzi D., Fondufe-Mittendorf Y., Martelli G., de Oliveira M.R., Montanaro R. (2021). Phenolic Compounds of Red Wine Aglianico del Vulture Modulate the Functional Activity of Macrophages via Inhibition of NF-κB and the Citrate Pathway. Oxidative Med. Cell. Longev..

[22] Xu D., Hu M.-J., Wang Y.-Q., Cui Y.-L. (2019). Antioxidant Activities of Quercetin and Its Complexes for Medicinal Application. Molecules.

[23] Chiummiento L., Funicello M., Lopardo M.T., Lupattelli P., Choppin S., Colobert F. (2012). Concise Total Synthesis of Permethylated Anigopreissin A, a New Benzofuryl Resveratrol Dimer. Eur. J. Org. Chem..

[24] Convertini P., Tramutola F., Iacobazzi V., Lupattelli P., Chiummiento L., Infantino V. (2015). Permethylated Anigopreissin A inhibits human hepatoma cell proliferation by mitochondria-induced apoptosis. Chem. Biol. Interact..

[25] de Oliveira M.R., Nabavi S.M., Braidy N., Setzer W.N., Ahmed T., Nabavi S.F. (2016). Quercetin and the mitochondria: A mechanistic view. Biotechnol. Adv..

[26] Kamiloglu S., Sari G., Ozdal T., Capanoglu E. (2020). Guidelines for cell viability assays. Food Front..

[27] Livak K.J., Schmittgen T.D. (2001). Analysis of relative gene expression data using real-time quantitative PCR and the 2(-Delta Delta C(T)) Method. Methods.

[28] Wojtala A., Bonora M., Malinska D., Pinton P., Duszynski J., Wieckowski M.R. (2014). Methods to monitor ROS production by fluorescence microscopy and fluorometry. Methods Enzym..

[29] Plaitakis A., Metaxari M., Shashidharan P. (2000). Nerve Tissue-Specific (*GLUD2*) and Housekeeping (*GLUD1*) Human Glutamate Dehydrogenases Are Regulated by Distinct Allosteric Mechanisms. J. Neurochem..

[30] Kim T.K. (2015). T test as a parametric statistic. Korean J. Anesth..

[31] Kim T.K. (2017). Understanding one-way ANOVA using conceptual figures. Korean J. Anesth..

[32] McIlwain D.R., Berger T., Mak T.W. (2013). Caspase functions in cell death and disease. Cold Spring Harb. Perspect. Biol..

[33] Boatright K.M., Salvesen G.S. (2003). Mechanisms of caspase activation. Curr. Opin. Cell Biol..

[34] Lopez J., Tait S.W.G. (2015). Mitochondrial apoptosis: Killing cancer using the enemy within. Br. J. Cancer.

[35] Khodapasand E., Jafarzadeh N., Farrokhi F., Kamalidehghan B., Houshmand M. (2015). Is Bax/Bcl-2 ratio considered as a prognostic marker with age and tumor location in colorectal cancer?. Iran. Biomed. J..

[36] Tsujimoto Y. (1998). Role of Bcl-2 family proteins in apoptosis: Apoptosomes or mitochondria?. Genes Cells.

[37] Agnello M., Morici G., Rinaldi A.M. (2008). A method for measuring mitochondrial mass and activity. Cytotechnology.

[38] Bunik V., Artiukhov A., Aleshin V., Mkrtchyan G. (2016). Multiple Forms of Glutamate Dehydrogenase in Animals: Structural Determinants and Physiological Implications. Biology.

[39] Rivera S., Azcón-Bieto J., López-Soriano F.J., Miralpeix M., Argilés J.M. (1988). Amino acid metabolism in tumour-bearing mice. Biochem. J..

[40] DeBerardinis R.J., Mancuso A., Daikhin E., Nissim I., Yudkoff M., Wehrli S., Thompson C.B. (2007). Beyond aerobic glycolysis: Transformed cells can engage in glutamine metabolism that exceeds the requirement for protein and nucleotide synthesis. Proc. Natl. Acad. Sci. USA.

[41] Li Y., Xu S., Li J., Zheng L., Feng M., Wang X., Han K., Pi H., Li M., Huang X. (2016). SIRT1 facilitates hepatocellular carcinoma metastasis by promoting PGC-1α-mediated mitochondrial biogenesis. Oncotarget.

[42] Sullivan L.B., Martinez-Garcia E., Nguyen H., Mullen A.R., Dufour E., Sudarshan S., Licht J.D., Deberardinis R.J., Chandel N.S. (2013). The proto-oncometabolite fumarate binds glutathione to amplify ROS-dependent signaling. Mol. Cell.

[43] Infantino V., Santarsiero A., Convertini P., Todisco S., Iacobazzi V. (2021). Cancer Cell Metabolism in Hypoxia: Role of HIF-1 as Key Regulator and Therapeutic Target. Int. J. Mol. Sci..

[44] Sun R.C., Denko N.C. (2014). Hypoxic regulation of glutamine metabolism through HIF1 and SIAH2 supports lipid synthesis that is necessary for tumor growth. Cell Metab..

[45] Alberghina L., Gaglio D. (2014). Redox control of glutamine utilization in cancer. Cell Death Dis..

[46] Granado-Serrano A.B., Martín M.A., Bravo L., Goya L., Ramos S. (2006). Quercetin Induces Apoptosis via Caspase Activation, Regulation of Bcl-2, and Inhibition of PI-3-Kinase/Akt and ERK Pathways in a Human Hepatoma Cell Line (HepG2). J. Nutr..

[47] Salama Y.A., El-Karef A., El Gayyar A.M., Abdel-Rahman N. (2019). Beyond its antioxidant properties: Quercetin targets multiple signalling pathways in hepatocellular carcinoma in rats. Life Sci..

