# Supplementary material for: Mitochondria-Mediated Apoptosis of HCC Cells Triggered by Knockdown of Glutamate Dehydrogenase 1: Perspective for Its Inhibition through Quercetin and Permethylated Anigopreissin A
Source: Biomedicines. 2021 Nov 11;9(11):1664. doi: 10.3390/biomedicines9111664 (PMC8615521; doi:10.3390/biomedicines9111664)
Supplement: Supplementary file 1 [file biomedicines-09-01664-s001.zip › biomedicines-1406716-supplementary.pdf]

Research Design

1. Hypothesis

1.1. Role of hGDH1 in liver cancer metabolism

2. Experimental research design

2.1. Omics analyis of hGLUD1 gene

2.1.1. Literature and database analysis

2.2. hGLUD1 gene silencing

2.3. hGDH1 activity inhibition

3. Data collection

3.1. Cell proliferation

3.2. Caspase and DNA fragmentation analysis

3.3. Mitochondrial alteration and apoptosis (mitochondrial mass, mitochondrial membrane potential, mitochondrial superoxide)

3.4. GDH1 activity assay with or without inhibitors

4. Data analysis

4.1. Statistical analysis

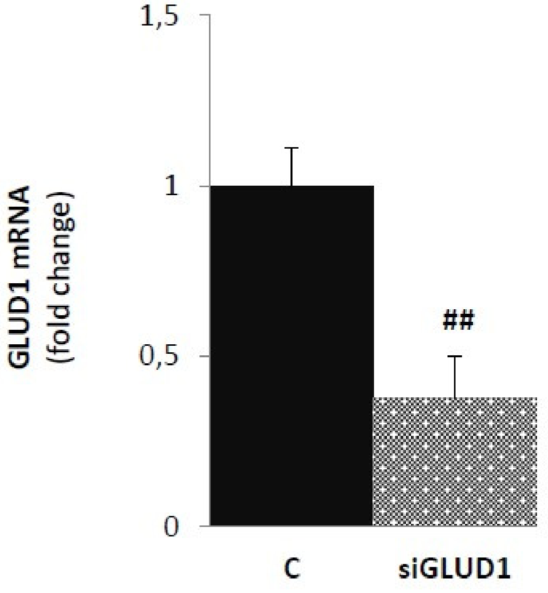

Figure S1. GLUD1 gene silencing in HepG2 cells. HepG2 cells were transfected with siRNA targeting human GLUD1 (siGLUD1) or control scramble siRNA (C) and used to quantify GLUD1 mRNA. Means ± S.D. of four replicate independent real-time PCR experiments are shown..
